# The Relationship between Lung Inflammation and Aerobic Threshold in Standardbred Racehorses with Mild-Moderate Equine Asthma

**DOI:** 10.3390/ani10081278

**Published:** 2020-07-27

**Authors:** Luca Stucchi, Elena Alberti, Giovanni Stancari, Bianca Conturba, Enrica Zucca, Francesco Ferrucci

**Affiliations:** 1 Equine Sports Medicine Laboratory “Franco Tradati”, Department of Health, Animal Science and Food Safety, Università degli Studi di Milano, via Celoria 10, 20133 Milano, Italy; luca.stucchi@unimi.it (L.S.); elena.alberti@unimi.it (E.A.); giovanni.stancari@unimi.it (G.S.); 2 Veterinary Teaching Hospital, Equine Medicine Unit, Università degli Studi di Milano, via Università 6, 26900 Lodi, Italy; bianca.conturba@unimi.it

**Keywords:** equine asthma, bronchoalveolar lavage, poor performance, lactate threshold

## Abstract

**Simple Summary:**

Horses can be affected by equine asthma, a disease that shares several similarities with human asthma. Young racehorses can be affected by a mild form of equine asthma, which can have a negative impact on racing performance. In this study, we evaluated the relation between the increase in inflammatory cells in the bronchoalveolar lavage fluid of horses affected by poor performance due to equine asthma and the speed at which the horse blood lactate reaches the value of 4 mmol/L, which is a parameter of athletic capacity. With this aim, we evaluated the results of a treadmill test with blood lactate analysis of 30 horses that had a bronchoalveolar lavage fluid cytology consistent with a mild form of equine asthma. The results showed a significant relation between the increase in the differential count of neutrophils in bronchoalveolar lavage and the decrease in the speed at 4 mmol/L of lactate. This confirm the negative impact of neutrophilic lung inflammation in the presence of equine asthma on athletic capacity and, consequently, on racing performance.

**Abstract:**

Mild–moderate equine asthma (MEA) is a common disease of young racehorses characterized by inflammation of the lower airways diagnosed by cytological examination of bronchoalveolar lavage fluid (BALF), and it is considered an important cause of poor performance. The most common parameter of athletic capacity associated with horse performance is the speed at 4 mmol/L of lactate (VLA4). The aim of the present work was to evaluate the relation between the different BALF inflammatory cells and VLA4 in racehorses affected by MEA. A population of Standardbred racehorses that underwent an accurate protocol for poor performance evaluation was collected for a retrospective study. Horses with any other alteration potentially influencing performance were excluded, thus considering only subjects with BALF cytology consistent with MEA. A sample of 30 horses (3.4 ± 1.0 years old) was selected. The relationship between BALF inflammatory cell differential count and VLA4 was evaluated by means of linear regression. Statistical analysis showed a significant relation (*p* = 0.015, r^2^ = 0.19) between the increase in the differential count of neutrophils in BALF and the decrease in VLA4. The results obtained suggested that the accumulation of neutrophils in the airways of MEA horses may have a direct impact on athletic capacity, possibly due to impaired alveolar blood-gas exchanges during strenuous exercise.

## 1. Introduction

Horses can be affected by equine asthma, an inflammatory non-infectious chronic lower airway disorder that shares several similarities with human asthma [1]. The mild–moderate form of equine asthma (MEA) is characterized by occasional coughing, tracheal mucus accumulation and decreased performance [2]. Horses of any age and breed could present MEA, even though young racehorses seem to be more commonly affected [3,4]. Diagnostic confirmation can be obtained by endoscopic examination of the airways, that exhibits an excess of tracheobronchial mucus, and by cytological examination of the bronchoalveolar lavage fluid (BALF), that reveals a mild increase in neutrophils, eosinophils, and/or metachromatic cells [2]. Recently, some authors suggested also the use of certain blood biomarkers (i.e., haptoglobin, secretoglobin, surfactant protein D) as diagnostic aids for horses with lower airway inflammation [5]. MEA differs from severe equine asthma (SEA, or “heaves”) due to the absence of respiratory effort at rest and the lack of evidence of airflow limitation measured by means of conventional lung mechanics [2].

MEA has been recognized as a cause of poor performance by several studies [6,7,8,9]. Recently, some authors reported the association between the accumulation of neutrophils or mast-cells in horse lungs and reduced performance [10], and the presence of a reduced maximum oxygen consumption (VO_2_ max) in horses with lung inflammation [11].

Nevertheless, reduced performance can be the result of several different causes, and it is difficult to quantify in an objective way the lower performance capacity of a horse. Some studies tried to calculate the lower level of performance on the basis of rider or trainer opinion, with the help of semiquantitative scores [12,13]. One technique that allows assessing in a quantitative way the athletic capacity of the horses is the calculation of some parameters based on the measurement of lactic acid produced during exercise. The most common parameter of aerobic capacity of the horse is the speed at which the blood lactate reaches the value of 4 mmol/L (VLA4). This value represents the threshold at which the horse shifts from aerobic to anaerobic metabolism [14]. Several studies reported that a higher VLA4 is associated with a better performance. In fact, VLA4 has been associated with some racing parameters [15], with racing time [16] and with racing performance [17].

The hypothesis was that as MEA is a cause of poor performance, the lower airway inflammation could be associated with a reduced lactate threshold. With these premises, the aim of the present work was to evaluate the relationship between the increase in different BALF inflammatory cells and the values of VLA4 in racehorses affected by MEA, using a retrospective study design.

## 2. Materials and Methods

### 2.1. Selection of the Sample

Subjects were selected retrospectively from a large population of Standardbred racehorses in training that were referred to the Equine Medicine Unit of the Veterinary Teaching Hospital of the University of Milan for poor performance evaluation. All horses were in their racing season and in full training.

All horses underwent a complete diagnostic protocol that included the following:

- Collection of history;
- Complete clinical examination with lameness evaluation;
- Laboratory evaluation (blood count, biochemistry and blood gas analysis);
- Electrocardiogram;
- Thoracic ultrasound (and cardiac evaluation in the presence of any murmur);
- Incremental treadmill test with plasma lactate analysis and Holter registration;
- Dynamic endoscopy of the upper airways on treadmill;
- Endoscopy of the airways with collection of BALF;
- Gastroscopy.

Horses with any other alteration (i.e., the presence of lameness, the presence of systemic illness, alteration in blood count or biochemistry, the presence of any cardiac arrhythmia or valvar alteration, alterations in the upper airways, the presence of equine gastric ulcer syndrome) potentially influencing performance were excluded, thus allowing us to consider only subjects with a cytological differential count of BALF consistent with MEA, on the basis of the following criteria [18]:

- Neutrophils > 5% and/or;
- Mast-cells > 2% and/or;
- Eosinophils > 1%.

For the present study no ethical approval has been requested, as all the procedures were performed on clinical horses with diagnostic purpose and included informed owner consent for the use of clinical data.

After application of inclusion criteria, 30 horses were selected (18 males, 8 females and 4 geldings) with an average age of 3.4 ± 1.0 years **.**

### 2.2. Incremental Treadmill Test, Blood Sample Collection and Processing and Calculation of VLA4

Each horse underwent an incremental high-speed treadmill test (Sato I, Uppsala, Sweden). Two days before the test, the horses were conditioned to the treadmill with two daily sessions, and the test was performed on the third day. The belt was inclined with a slope of 5% and the protocol consisted of a warm up of 4 min walk (1.5 m/s) and 3 min trot (6 m/s), followed by 1 min phases increasing the speed of 1 m/s for each stage, until the horse was no longer able to maintain the treadmill speed (maximal speed 12 m/s). Cool down was achieved by 30 min walk with 0% slope [19].

Blood samples during the test were taken without stopping the treadmill with the aid of a 14 G Teflon venous catheter placed in the jugular vein connected to an extension tube. Blood samples were taken at rest, after the warm-up, just before increasing the speed at the end of each phase, and at 5, 10 and 30 min during the recovery phase. After collection, blood was transferred into tubes containing 10 mg of sodium fluoride and 2 mg of potassium oxalate for 1 mL of blood. Within 15 min, the samples were centrifuged (4000 g for 10 min) to obtain plasma, refrigerated (2–8 °C) and analyzed. Plasma lactate (mmol/L) was measured using the enzymatic colorimetric method with a lactate dry-fast kit for the automatic system (Uni Fast System II Analyzer, Sclavo, Italy) and reagents supplied by the manufacturer.

Data concerning the values of plasma lactate at each speed stage were collected on an electronic sheet (Microsoft Excel, Redmond, USA) and VLA4 was calculated for each horse with dedicated software (Lactate-E) [20]. This was calculated using inverse prediction by finding the speed corresponding to a lactate value equal to 4 mmol/L.

### 2.3. BALF Collection and Cytological Examination

The day after the treadmill test, a BALF sample was obtained by means of a flexible videoendoscope (Storz, Tuttlingen, Germany). The horses were sedated with 0.01 mg/Kg detomidine and restrained in stock. The endoscope, previously cleaned and disinfected with 2% glutaraldehyde solution, was passed from the nostril to the carina; here a 0.5% lidocaine solution was sprayed in order to avoid coughing reflex. Then, the endoscope was passed into the bronchial tree until it was wedged with the diameter of a single bronchus. A 450 mL 0.9% saline solution was then instilled and immediately aspired [21].

The sample was subsequently stored in ethylenediaminetetraacetic acid (EDTA) tubes and immediately processed. A few drops of pooled BALF were cytocentrifuged (Rotofix 32, Hettich Cyto System, Germany) at 500 rpm for 5 min. The slides were air dried, stained with May–Gruenwald–Giemsa and observed at 400× and 1000× for standard 400-cell leukocyte differential count [22].

### 2.4. Statistical Analysis

Statistical analysis was performed by means of a commercial software (Graphpad Prism, La Jolla, CA, USA). The relation between age and BALF cytology or VLA4, and the relation between BALF inflammatory cell differential count and the values of VLA4 were evaluated by means of linear regression. The effect of sex on BALF cytology and on VLA4 was evaluated by means of Kruskal–Wallis test. Statistical significance was set at *p* < 0.05.

## 3. Results

The average value of VLA4 obtained from the horses of the sample was 8.7 ± 1.0 m/s.

The differential count of the inflammatory cells in BALF of the sample showed an average value of 42.6 ± 8.9% for macrophages, 38.5 ± 12.7% for lymphocytes, 11.4 ± 9.3% for neutrophils, 2.7 ± 3.9% for eosinophils and 4.9 ± 2.0% for mast-cells.

**Figure 1:**
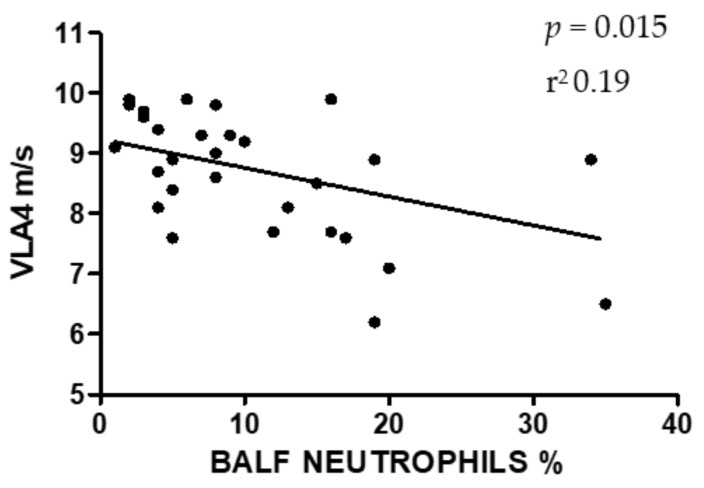
Results of linear regression between the percentage of bronchoalveolar lavage fluid (BALF) neutrophils and 4 mmol/L of lactate (VLA4).

**Figure 2:**
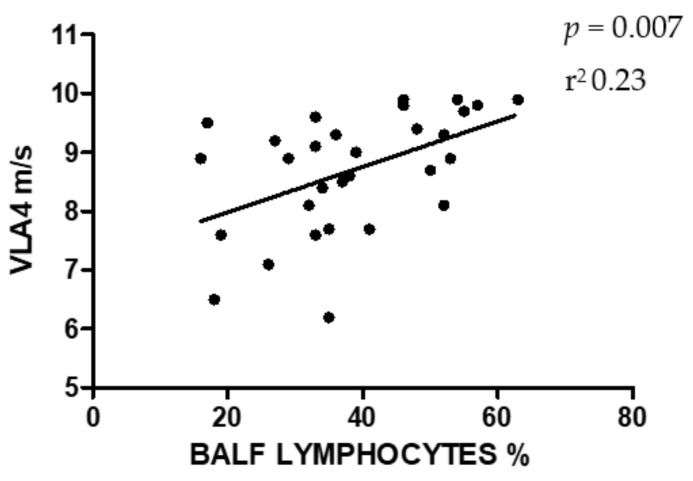
Results of linear regression between the percentage of BALF lymphocytes and VLA4.

Statistical analysis showed a significant relation (*p* = 0.015, coefficient of determination r^2^ = 0.19) between the increase in the differential count of neutrophils in BALF and the decrease in VLA4 (Figure 1), and a significant relation between the increase in the differential count of lymphocytes and the increase in VLA4 (*p* = 0.007, r^2^ = 0.23) (Figure 2). No relation was found between other cell types and VLA4. Moreover, the results showed no relation between age or sex on VLA4 or BALF differential count.

## 4. Discussion

The aim of the present study was to evaluate the relationship between the different inflammatory cell populations of the lower airways of Standardbred racehorses affected by MEA and their value of VLA4.

As all the horses admitted at our hospital for performance profiling underwent to the same protocol of clinical investigation, we could select the samples with the exclusion of all the horses affected by the other common causes of reduced performance, such as the presence of lameness, systemic infection or inflammation, dynamic upper airways obstruction, cardiac alteration, blood in the trachea after the exercise, rhabdomyolysis or equine gastric ulcer syndrome [23]. In fact, as poor performance can be the results of a combination of several factors, it is common that two or more diseases are present in the same horse, and the real cause of lower athletic capacity can be debatable. Thus, the reason that the sample number of the study being relatively small (30 subjects) is because horses in which MEA was the only disease potentially affecting performance were selected.

The selection of VLA4 as parameter of performance was driven by the number of studies published on this topic [15,16,17]. The limit of the use of VLA4 as a parameter of performance is that it can be affected by the level of training of the horse [24]. Horses of our sample, however, were in their racing season, in full training and it could be argued that they were at the same level of fitness.

In the literature, some studies report that the percentage of neutrophils increases with age, such as the value of VLA4 [12,25]. However, in our work, we found no of association between age and BALF differential cell count or VLA4. In our work, it is probable that the age range was too small (2–5 years old) to detect any effect of age on these parameters. While it has been reported that sex could have an influence on VLA4 [26], in our sample we did not find any difference for VLA4 related to sex, probably due to the small number of the sample group. In regard to the differential count of BALF, to our knowledge, no association between sex and cell percentage has been reported.

The weak, but significant relation between the increase in the percentage of neutrophils in BALF and the decrease in VLA4 is in accordance with data reported by previous studies [27,28], even if this is the first time that a proportional association between athletic capacity and neutrophilic lung inflammation is reported. It can be hypothesized that the accumulation of neutrophils in the airways of MEA horses induces edema and inflammation, which might affect alveolar blood–gas exchanges during strenuous exercise [27]. The oxygen transport is consequently reduced, causing a more pronounced exercise-induced hypoxemia [28], probably leading to a premature passage to the anaerobic metabolism. This could induce an early lactate accumulation, a lower VLA4 and a reduced athletic capacity.

It has also been demonstrated that the presence of tracheal mucus during endoscopy is associated with reduced performance [29], and some authors reported that tracheal mucus is directly related to the percentage of neutrophils [30]. In the present study we did not evaluate the relation between the endoscopic score and VLA4, thus, this association between tracheal mucus and VLA4 would be of great interest in future studies.

The significant relationship found between the increase in the percentage of lymphocytes and a better VLA4 is not, from our perspective, related to a biological reason but, instead, this association can be the result of the use of a differential count of inflammatory cells as a tool for diagnosis. In fact, due to the little variation in the relative count of macrophages, eosinophils or mast-cells, the increase in the percentage of lymphocytes can be the consequence of the decrease of the percentage of neutrophils, and it could have influenced the statistical result.

Finally, the percentage of mast-cells and eosinophils in BALF seemed to have no relation with VLA4. One recent study reported that the increase in mast-cells in BALF had a negative impact on performance [10], however, further studies are needed on this topic, perhaps selecting MEA horses with only eosinophilic or mast-cell inflammation.

## 5. Conclusions

In conclusion, the results obtained from the present study showed that the increase in the percentage of neutrophils in BALF of racehorses with poor performance affected by MEA is associated with a lower VLA4, confirming the role of MEA as a cause of reduced performance.
